# Integration of growth factor gene delivery with collagen‐triggered wound repair cascades using collagen‐mimetic peptides

**DOI:** 10.1002/btm2.10037

**Published:** 2016-10-19

**Authors:** Morgan A. Urello, Kristi L. Kiick, Millicent O. Sullivan

**Affiliations:** ^1^ Dept. of Chemical and Biomolecular Engineering University of Delaware Newark DE 19716; ^2^ Dept. of Material Science and Engineering University of Delaware Newark DE 19716

**Keywords:** collagen‐like peptides, collagen‐mimetic peptides, growth factor therapy, nonviral gene delivery, wound repair

## Abstract

Growth factors (GFs) play vital roles in wound repair. Many GF therapies have reached clinical trials, but success has been hindered by safety concerns and a lack of efficacy. Previously, we presented an approach to produce protein factors in wound beds through localized gene delivery mediated by biomimetic peptides. Modification of polyethylenimine (PEI) DNA polyplexes with collagen‐mimetic peptides (CMPs) enabled tailoring of polyplex release/retention and improved gene transfer activity in a cell‐responsive manner. In this work, CMP‐mediated delivery from collagen was shown to improve expression of platelet‐derived growth factor–BB (PDGF‐BB) and promote a diverse range of cellular processes associated with wound healing, including proliferation, extracellular matrix production, and chemotaxis. Collagens were pre‐exposed to physiologically‐simulating conditions (complete media, 37°C) for days to weeks prior to cell seeding to simulate the environment within typical wound dressings. In cell proliferation studies, significant increases in cell counts were demonstrated in collagen gels containing CMP‐modified polyplex versus unmodified polyplex, and these effects became most pronounced following prolonged preincubation periods of greater than a week. Collagen containing CMP‐modified polyplexes also induced a twofold increase in gel contraction as well as enhanced directionality and migratory activity in response to cell‐secreted PDGF‐BB gradients. While these PDGF‐BB‐triggered behaviors were observed in collagens containing unmodified polyplexes, the responses withstood much longer preincubation periods in CMP‐modified polyplex samples (10 days vs. <5 days). Furthermore, enhanced closure rates in an in vitro wound model suggested that CMP‐based PDGF‐BB delivery may have utility in actual wound repair and other regenerative medicine applications.

## 1 Introduction

Identification of the growth‐promoting activities of the secreted, signaling proteins known as growth factors (GFs) has inspired much anticipation about their potential in tissue repair applications, particularly in refractory wounds and other hard‐to‐heal tissues. These multifunctional and potent proteins play fundamental roles in a range of regenerative activities including regulation of cellular proliferation, chemotaxis, and extracellular matrix synthesis, with their activity often recognizable in the picomolar range.^1^, ^2^ For example, altered cell phenotypes and an aberrant extracellular environment in chronic wounds are factors recognized to reduce GF production, stability, accessibility, and activity, further complicating the already intricate reparative processes.^3^, ^4^, ^5^, ^6^, ^7^ Accordingly, multiple preclinical studies and industry sponsored trials have examined the efficacy of topical and sustained release GF formulations in chronic wounds.^6^, ^8^ In 1997, Becaplermin/Regranex (Systagenix; Skipton, U.K.), a topical platelet‐derived growth factor‐BB (PDBF‐BB) gel, became the first successful, FDA‐approved growth factor treatment for the treatment of diabetic foot ulcers (DFUs)^9^; however, existing GF therapies exhibit only modest clinical utility overall. Based on clinical trials, the FDA concluded that topically‐applied PDGF‐BB increased the number of healed DFU patients by less than 10%,^6^ and while the application of PDGF‐BB has been shown to augment wound repair in several human studies, many were never published due to a lack of efficacy.^9^, ^10^, ^11^, ^12^ Clinical failure has largely been blamed on incompatibilities of traditional GF therapies with the hostile, irregular chronic wound environment that limits GF penetration into the wound bed, causes rapid GF degradation due to elevated protease activation, and decreases cellular responses to GFs.^3^, ^5^, ^8^ Accordingly, extraphysiological, repetitive doses are typically required to achieve therapeutic effects. These dosing regimens increase the danger of GF toxicity, elevate treatments costs, and elevate the risk of substantial off‐target effects or even oncogenic responses.^3^, ^9^, ^13^, ^14^, ^15^ New approaches for creating healthy, GF‐rich wound beds are essential.

Based on the need for improved wound therapies, promising alternatives include GF gene delivery and the application of biocompatible matrices that can regulate multiple aspects of cell behavior through controlled presentation of extracellular cues. GF gene therapies offer exciting potential benefits for improved GF delivery due to their ability to foster localized, on‐demand GF production within the wound bed. In particular, gene‐based approaches to GF delivery better mimic endogenous repair responses by allowing host cells to orchestrate sustained GF expression, microlocalization, and activity, which are essential in chronic wound repair due to extended healing over months, spatiotemporal heterogeneity, and elevated protease activation. Because of these characteristics, GF gene therapies have exhibited increased efficacy in experimental wound models as compared with topical delivery approaches, with the capacity to achieve similar healing responses with orders of magnitude less GF (e.g., ∼2,000‐fold less GF expression than a typical topically‐applied dose).^16^, ^17^, ^18^, ^19^, ^20^, ^21^, ^22^, ^23^ These observed dosage reductions coupled with the spatiotemporal control achievable via promoter choice/vector design^24^, ^25^, ^26^ suggest that gene therapies may have exciting potential to create controllable, more effective, and less toxic approaches to deliver GFs. While clinical data on GF gene therapies are limited, localized gene therapy approaches show promise for improved safety and efficacy, and are among the most rapidly advancing gene therapies in clinical trials for diseases such as ocular disorders.^6^, ^19^, ^27^

In terms of delivery regimens, therapeutic DNA has been incorporated into biomaterial matrices designed to mediate, prolong, and enhance gene transfer while reducing potential off‐target and/or immune responses.^8^, ^19^, ^28^, ^29^, ^30^, ^31^, ^32^, ^33^, ^34^, ^35^, ^36^ In addition to providing enhanced gene stability and improved control over release, gene activated matrices (GAMs) provide a permissive environment that promotes cellular ingrowth, increases tissue deposition, triggers in situ production of GFs, and enhances cell health.^15^, ^28^, ^36^ In fact, the application of collagen‐based artificial skins, such as Apligraf (Organogenesis) and Dermagraft (Advanced Biohealing), has been shown to enhance chronic wound repair even in the absence of incorporated GFs; however, the incidence of complete closure after a therapeutic trial with engineered skin remained close to 50%, highlighting the need for improved bioactivity.^8^ The synergistic effects in wound repair between biomaterials and GF‐encoding genes have been demonstrated in numerous studies, such as when collagen‐embedded *PDGF‐B* DNA was shown to increase the formation of new granulation tissue by up to 52% and re‐epithelization by up to 34%, as compared to collagen alone, in a dermal ulcer model in rabbits. The same materials stimulated a more than fourfold increase in cell repopulation over a 10‐day period in an ex vivo human gingival defect repair model.^8^, ^37^

Additional studies demonstrate the clear advantages for natural and synthetic GAMs in controlling gene transfer efficacy, with some approaches reporting detectable gene expression in vivo over a few weeks via diffusion‐ and/or degradation‐controlled retention/release of entrapped plasmids or polyplexes.^30^, ^38^, ^39^, ^40^ Furthermore, improvements in spatial and temporal control over the delivery of DNA from GAMs have been achieved through the immobilization of DNA onto scaffolds through better defined interactions, such as biotin‐avidin or antigen‐antibody binding.^32^, ^41^, ^42^, ^43^, ^44^ For instance, biotinylation of PEI DNA polyplexes increased retention onto avidin‐modified collagen by as much as 30%, resulting in a twofold increase in transfection efficiency compared to that observed in collagen encapsulating unmodified PEI DNA polyplexes.^31^ However, while current gene‐based therapeutics are very promising, they often have failed in translation due to continued concerns of off‐target and immune responses, as well as inefficiencies in gene transfer efficacy in protein/serum‐rich environments.^19^, ^45^, ^46^, ^47^, ^48^ Moreover, the majority of existing GAM technologies are unfit for many tissue repair applications due to the complexity of the healing process, which can involve extended healing periods over months and multiple out‐of‐phase healing cascades occurring simultaneously within repair sites.

In our prior studies, a novel approach with the potential to overcome these issues through application of collagen‐mimetic peptides (CMPs) in gene delivery was demonstrated. CMPs have a natural affinity for collagen driven by a reversible strand‐invasion process that can be tailored with relative ease by altering CMP sequence and molecular weight. This unique ability has been exploited to modify extracted collagens in vitro,^34^, ^49^, ^50^, ^51^, ^52^, ^53^ as well as to target and bind remodeling collagens in vivo,^49^, ^54^, ^55^ using various CMP‐linked cargoes such as GFs. Our labs were the first to use CMPs to modify collagen with DNA. Specifically, CMP display on DNA‐polyethylenimine (PEI) polyplexes was shown to have the capacity to improve control over both the extent and duration of gene expression. Through varying CMP display, DNA release/retention was tailored for over a month, two times longer than the retention/release periods of unmodified polyplexes. CMP‐modification also maintained polyplex activity in serum‐supplemented media for up to 2 weeks, in contrast with most gene delivery approaches which report losses to nuclease degradation within hours.^34^, ^56^ Additionally, we demonstrated the novel ability to “hijack” collagen remodeling,^56^ a process that occurs in excess in the protease‐rich chronic wound environment.^3^, ^8^, ^9^ Whereas previous studies have utilized proteolytically‐sensitive materials to synchronize cell invasion with therapeutic release, the reversible, serum‐stable nature of the CMP‐collagen interaction allowed for continued association with collagen fragments, confirmed through colocalization microscopy studies. The alteration in polyplex composition resulted in enhanced polyplex activity linked to an increased capacity to preserve DNA integrity in the presence of serum and an increase in caveolar uptake, a pathway linked to high efficiency gene transfer. Furthermore, the benefits of using collagen remodeling as a driver for gene release and activity were confirmed in a more complicated, in vivo model in which transgene expression was localized and extended from 3 to over 20 days.^56^ This versatile approach, which capitalizes on collagen remodeling, has the potential to efficiently augment any collagen‐containing device with gene expression and has tremendous potential for overcoming non‐viral gene delivery obstacles in multiple regenerative medicine applications.

In this work, our objective was to demonstrate the benefits of CMP gene delivery for GF expression during wound healing. For this purpose, we chose PDGF‐BB as a target GF due to its well established ability to effect a diverse range of cellular processes associated with wound healing, including proliferation, extracellular matrix production, and chemotaxis.^8^, ^19^, ^37^, ^43^, ^57^, ^58^, ^59^ Our studies demonstrated that CMP display on PEI polyplexes encoding PDGF‐BB significantly enhanced polyplex activity, even after prolonged exposure to physiologically‐simulating conditions mimicking the wound environment. PDGF‐BB levels were up to fourfold greater in CMP‐modified samples, versus unmodified samples, and maintained up to a threefold increase in expression even after the gene‐modified collagen scaffolds were exposed to serum for 7 days at 37°C. Various desirable cell repair behaviors were also enhanced by CMP‐modification. Cell counts were increased by up to 75% in CMP scaffolds, and CMP‐induced differences in proliferation remained significant even after a 10‐day serum preincubation. Collagen remodeling and cell migration were also enhanced by CMPs, as highlighted through collagen contraction assays and cell migration studies. Moreover, we demonstrated promising capacity for the CMP‐modified DNA/collagen gels in wound scaffold applications, based on increased cell densities and accelerated migration in collagen‐based, 3‐D wound models. Defects treated with CMP‐modified collagens reached approximately 90% wound closure after 10 days of treatment, whereas wound closure never exceeded 40% using scaffolds containing unmodified polyplex. Moreover, collagen scaffolds supplemented with recombinant PDGF‐BB (rPDGF‐BB) at levels comparable to the maximum expression levels exhibited no differences in cell invasion/proliferation from collagen alone; in fact, rPDGF‐BB levels had to be increased by an order of magnitude to achieve similar bioactivity as in CMP‐modified scaffolds. The increase in gene stability and improved expression associated with CMP‐based gene delivery may translate in vivo, aiding in multiple aspects of wound repair. Furthermore, our findings stress the advantages of GF gene therapies for providing substantial reductions in GF exposure and thereby reducing concerns with both cost and safety. These advantages highlight the potential of CMP‐triggered gene delivery to enhance numerous collagen‐based materials through improved non‐viral gene delivery regimens.

## 2 Material and methods

### 2.1 Materials

Type I bovine collagen was purchased from Advanced BioMatrix (San Diego, CA) and pCMV‐PDGF‐BB plasmid was purchased from Origene Technologies, Inc. (Rockville, MD). pDNA was amplified in NEB 5‐α electrocompetent *E. coli* purchased from New England Biolabs and purified from bacterial culture using a Qiagen Megaprep Kit (Valencia, CA), following the manufacturer's protocols. The Mouse/Rat PDGF‐BB Quantikine enzyme‐linked immunosorbent assay (ELISA) kit was purchased from R&D Systems (Minneapolis, MN). Fmoc‐protected amino acids were purchased from Anaspec (Fremont, CA). H‐Rink amide ChemMatrix® resin was purchased from PCAS Biomatrix (Quebec, Canada). O‐Benzotriazole‐ N,N,N′,N′‐tetramethyl‐uronium‐hexafluoro‐phosphate (HBTU) was purchased from Novabiochem (San Diego, CA). High performance liquid chromatography (HPLC)‐grade N,N‐dimethyl formamide (DMF), acetonitrile, trifluoroacetic acid (TFA), CellTracker^TM^ Deep Red, and cell culture reagents, including Dulbecco's modified Eagle's medium (DMEM), Dulbecco's phosphate buffered saline (PBS), penicillin‐streptomycin (P/S), and trypsin were purchased from Fisher Scientific (Fairlawn, NJ). Fetal bovine serum (FBS) was purchased from Corning (Manassas, MA). Collagenase I was purchased from Worthington Biochemical Corp (Lakewoord, NJ). Piperidine, 4‐methylmorpholine, all cleavage cocktail components, and branched PEI (25 kDa) were purchased from Sigma‐Aldrich (St. Louis, MO). The Oris^TM^ cell migration assay kit was purchased from Platypus Technologies (Madison, WI).

### 2.2 Preparation of modified collagen gels

The CMP [GPP: (GPP)_3_GPRGEKGERGPR(GPP)_3_] used in prior studies^34^, ^60^, ^61^ was synthesized using Fmoc solid phase peptide synthesis and purified using reverse phase‐HPLC. GPP was conjugated to PEI using Michael‐type addition chemistry and the conjugate (GPP‐PEI) was used to prepare GPP‐modified polyplexes as previously described.^34^ Using a variation of well‐established polyplex formation protocols,^62^, ^63^ equivolumetric solutions of PEI and DNA in 20 mM HEPES buffer (pH 6.0) were mixed to produce a final solution with an amine to phosphate ratio (N:P) ratio of 10. To incorporate GPP, the GPP‐PEI conjugate was preincubated at 50°C for 30 min to prevent triple‐helical hybridization of GPP, and a specified percent of PEI used to create the polyplex was replaced with the GPP‐PEI. Collagen gels with GPP‐immobilized or encapsulated unmodified polyplexes were prepared by re‐suspending dehydrated polyplex in neutralized type I bovine collagen‐solution (4 mg/ml, pH 7.4) as previously described.^34^ After a 3 hr incubation on ice to allow bubbles to settle and enable GPP‐collagen hybridization, the solution was allowed to gel overnight at 37°C.

### 2.3 Quantification of PDGF‐BB expression in modified collagens

DNA/collagen gels were prepared with 500 µl of DNA/collagen solution in 8 well plates (0.8 cm^2^ surface area/well). Gels were incubated at 37°C overnight to allow gelation, after which 200 µl of complete medium (DMEM with 10% FBS and 1% P/S) was added. The gels were then preincubated in complete medium at 37°C and 5% CO_2_ for a specified time period ranging from 0 to 14 days to simulate physiological conditions. MMPs and other wound‐relevant proteases were purposefully excluded from the pre‐treatment step based on wound environment studies that demonstrate localization of proteases in the expressing cells’ microenvironment coinciding with the wound edge or adjacent tissues.^64^ Subsequently, gels were washed with PBS and DMEM and 20,000 NIH/3T3 cells were seeded per well. Cells were cultured under the same conditions as the preincubation. After a 4‐day culture, a sandwich ELISA and a direct ELISA were used to determine PDGF‐BB concentrations in the conditioned media and in the gels, respectively. PDGF‐BB remaining in the gels was recovered through a 72‐hr incubation with 500 µl of extraction buffer (10 mg/ml heparin, 2% BSA, 2M sodium chloride, and 0.01% Triton‐X in PBS) at 4°C. Released PDGF‐BB was quantified using a commercially available Quantikine ELISA kit in a 96‐well plate format, according to manufacturer's instructions. A seven‐point standard curve was used to quantify the concentrations, spanning a range from 0 to 2,000 ng/ml PDGF‐BB. Each sample was read twice with a Glomax Multimodal Plate reader (Sigma), and the average of the two readings was used to calculate the concentration of PDGF‐BB in the sample. PDGF‐BB still remaining in the gel was quantified through use of a direct ELISA. After removal of the extraction buffer, the gels were gently washed twice with PBST (PBS supplemented with 0.05% Tween‐20, 5 min per wash) and incubated with the anti‐mouse/rat PDGF‐BB antibody conjugate supplied in the Quantikine ELISA kit (antibody specific for PDGF‐BB conjugated to horseradish peroxidase [HRP]) for 2 hr at room temperature. Following two 5‐min PBST washes, 300 µl of a 1:1 solution of color reagent A (hydrogen peroxide) and color reagent B (tetramethylbenzidine) were added. Colored solution was removed from each gel after 30 min and a stop solution was added. The Glomax Multimodal Plate reader (Sigma) was then used as described above to determine PDBF‐BB concentrations using solutions collected from an empty collagen control gel as a background measurement.

### 2.4 Quantification of proliferation

DNA/collagen gels were prepared and preincubated for periods ranging from 0 to 14 days as described above. Four days after seeding cells (20,000 NIH/3T3 cells per well), gels/cells were imaged using a Leica DMI6000 B inverted microscope (Wetzlar, Germany), and image analysis tools in ImageJ (National Institutes of Health) were used to count the number of cells per field of view. Cells were subsequently recovered from the gels through use of a collagenase (0.1 U/ml PBS)/dispase® (0.8 U/ml PBS) digestion solution, as well as gentle pipetting to break the gel apart. After a 45‐minute digestion at 37°C, the total cell count was determined using a hemocytometer and compared to the number of cells initially seeded. Samples were analyzed at 4 days to provide time for cellular invasion, transfection, and proliferation while excluding the effects of cellular confluency on cellular behavior.

### 2.5 Contraction assay

DNA/collagen gels were prepared and preincubated as described above. After a 0‐ to 10‐day preincubation, 20,000 cells were seeded onto the gels. Three days after seeding the cells, gel height was determined by analyzing images taken of the gels with a camera, using a ruler tool in ImageJ. To account for gel irregularities, the gel height for a given gel was determined by averaging the heights measured at 5 set‐points in the gel (one point at the center of gel; 2 points at the gel edges, and 2 points equidistant from the prior points). The average gel height reported for each sample was based upon the average of individual gel heights from 5 different samples.

### 2.6 Collagen bi‐layer cellular migration study

**Figure 1:**
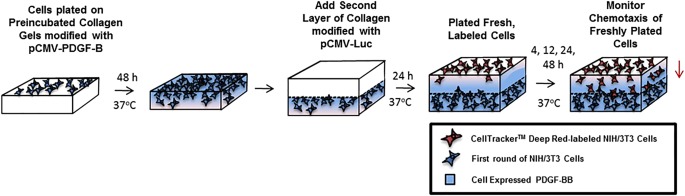
Schematic of collagen bi‐layer migration study. NIH/3T3 cells were seeded onto a layer (layer 1) of collagen containing polyplex encoding for PDGF‐BB. Layer 1 was preincubated in complete culture media containing 10% serum prior to cell plating. After a 48 hr culture of the cells on layer 1, a second layer (layer 2) of collagen (containing pCMV‐Luc) was added. Twenty‐four hours after the addition of layer 2, additional NIH/3T3 cells were plated on top of layer 2 and migration in response to the cell‐expressed PDGF‐BB (in layer 1) was monitored.

Layered DNA/collagen gels containing a gradient in cell‐expressed PDGF‐BB were produced through a multi‐step procedure (Figure 1). First, 200 µl DNA/collagen gels containing PDGF‐BB‐encoding polyplexes were prepared and preincubated in complete media [(10% FBS, 1% P/S) at 37°C and 5% CO_2_], as described, to simulate physiological conditions. After a 0‐ to 10‐day preincubation period, 20,000 NIH/3T3 cells were seeded onto the collagen gels and cultured for 2 days under the same conditions as the preincubation to allow cell invasion and transfection. Media was subsequently removed from the gels and a second layer of collagen containing luciferase‐encoding polyplex (100 µl of collagen/DNA solution) was added atop each cellularized gel. Following gelation at 37°C for 2 hr, complete media was added to the bi‐layer collagen gels. After allowing an additional 24 hr for a gradient in PDGF‐BB expression to form, 5,000 fresh NIH/3T3 cells (labeled with CellTracker^TM^ Deep Red) were plated onto each gel. These cells were prelabeled with the CellTracker^TM^ Deep Red dye via the manufacturer's protocol to enable rapid tracking of cell migration, and the cells were plated at a low density (∼6,250 cells/cm^2^) to minimize the effects of cell‐induced contraction.^65^, ^66^ A 75% reduction in cell seeding densities lessened contraction in collagen gel height from approximately 16 to 5% over 48 hr (data not shown) and migration was monitored using a Leica DMI6000 B inverted microscope (Wetzlar, Germany). All image analysis was performed using ImageJ.

### 2.7 In vitro wound model

3‐D in vitro defect wounds were constructed using an Oris^TM^ cell migration assay kit. A physical “stopper” barrier was placed in the center of each well of a 96‐well plate. Twenty microliters of collagen solution containing 100,000 NIH/3T3 cells/ml were added to either side of the stopper (total of 40 µl per well) to ensure the collagen/cell mixture was spread evenly around the stopper. After allowing gelation at 37°C for 1 hr, the stopper was removed, creating uniform, cell‐free defects at the center of each well (diameter = 2 mm). Twenty microliters of DNA/collagen solution encoding PDGF‐BB, prepared as described previously, was immediately added to the defect. In the 20%/50% GPP‐modified polyplex samples a ratio of 8:2 (mass of DNA in the 20% vs. 50% GPP‐modified polyplex) was used. After allowing gelation to occur for 1 hr at 37°C, complete media was added atop each gel and cell invasion into each defect was monitored via Calcein AM staining (5 µM Calcein AM in Opti‐MEM). Defect/wound “closure” was analyzed using the thresholding function in the ImageJ MRI Wound Healing Tool (NIH), with the new wound edge defined as the point when the average cell density/fluorescence in the defect matched the average cell density/fluorescence in the area surrounding the defect. Percent wound closure was defined as the fractional area of the defect that has the same cell density as the area surrounding the defect.

## 3 Results

### 3.1 Quantification of PDGF‐BB expression in modified collagens

**Figure 2:**
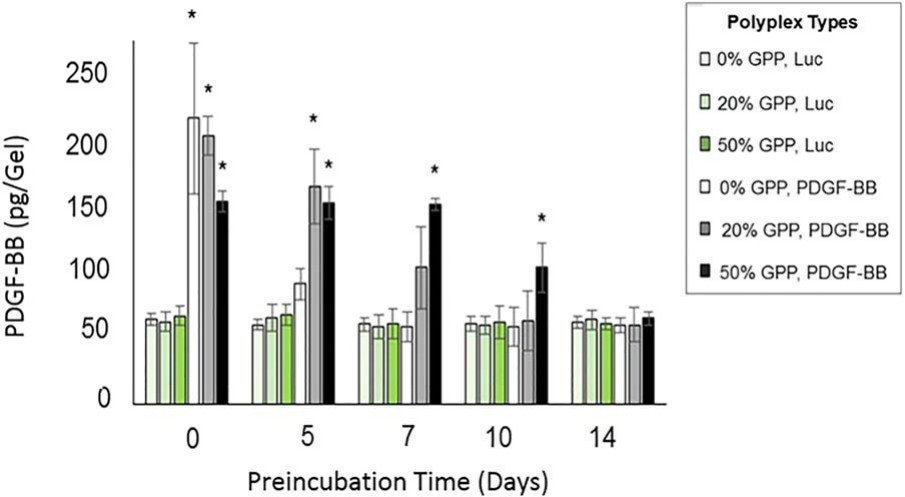
Cellular expression of PDGF‐BB was analyzed 4 days after NIH/3T3 cells were plated onto collagen gels containing polyplexes. The data represent the amounts of collagen‐bound PDGF‐BB in each gel, as assessed by ELISA. PDGF‐BB levels in the conditioned media were insignificant and hence not included in the analysis. The data represent the mean ± standard deviation (SD) in four separately prepared and analyzed samples. * denotes a statistically‐significant difference (*p* < .05) relative to the luciferase‐encoding controls

To quantify cell‐expressed PDGF‐BB in the modified‐collagens, both direct and indirect sandwich ELISA assays were used. No significant differences in the PDGF‐BB concentrations were detected in conditioned media collected from any of the samples, based upon sandwich ELISA measurements; however, significant differences between samples were detected when the levels of collagen‐bound PDGF‐BB were analyzed by release from collagen via extraction buffer and direct ELISA (Figure 2). In particular, when samples were not preincubated, collagen‐bound PDGF‐BB levels were elevated in all samples containing polyplex encoding for PDGF‐BB relative to samples containing polyplex encoding for luciferase. In the non‐preincubated samples, the highest levels of expression were observed in the PDGF‐BB‐encoding samples with unmodified and 20% GPP‐PEI/total PEI polyplex, which exhibited over a fourfold increase in expression relative to the luciferase controls. Levels of expression in the 50% GPP‐PEI/total PEI PDGF‐BB sample were also elevated in these samples, with a nearly threefold increase in expression. In the unmodified polyplex samples, the level of expression rapidly decreased when the gels were exposed to wound‐mimetic conditions, through preincubation of the gels with serum solutions at 37°C. PDGF‐BB expression decreased by more than 60% after a 5‐day serum preincubation in the unmodified polyplex samples, and at this time point, the expression levels ceased to be higher than those overserved in the luciferase controls. Expression in the GPP‐modified samples also decreased as a function of preincubation time; however, the rate of decrease was substantially slower. After a 5‐day preincubation, expression decreased in the 20% GPP‐PEI/total PEI samples by only 20%, and the expression levels decreased 55% after a 7‐day preincubation, relative to the non‐preincubated samples. PDGF‐BB expression in the 20% GPP‐PEI/total PEI samples ceased to be greater than the luciferase‐encoding control samples after preincubation periods over 7 days. The 50% GPP‐PEI/total PEI samples exhibited an increased longevity in gene transfer activity when exposed to physiologically‐simulating conditions. These samples maintained consistent levels of expression that were approximately threefold greater than the luciferase‐encoding controls for up to a 7‐day preincubation period. The samples exhibited significantly elevated levels of PDGF‐BB relative to the controls for up to a 10‐day preincubation.

### 3.2 Quantification of PDGF‐BB‐mediated cellular proliferation

**Figure 3:**
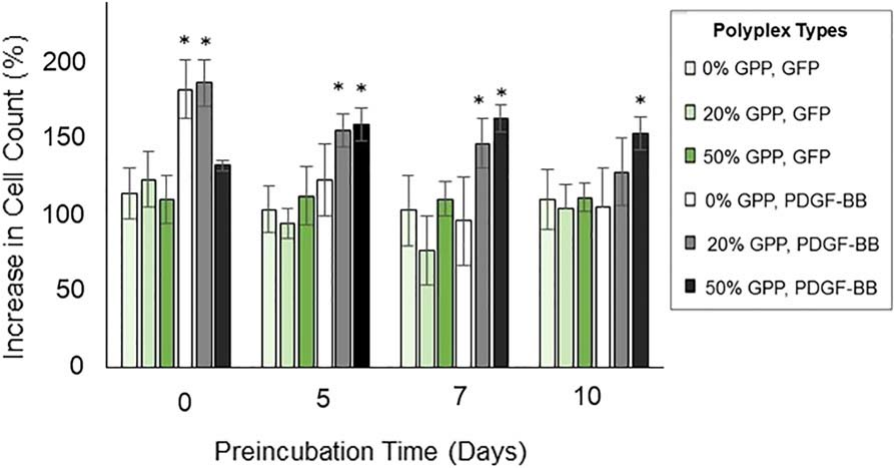
Cellular proliferation in preincubated collagen gels containing polyplexes, 4 days after NIH/3T3 cells were seeded onto the gels. Proliferation was analyzed to determine the mitogenic activity of cell‐expressed PDGF‐BB. The data represent the mean ± SD of the increase in cell count, relative to the number of cells initially seeded, as assessed with a hemocytometer after cell recovery from the gels, in four separately prepared and analyzed samples. * denotes a statistically‐significant difference (*p* < .05) relative to the GFP‐encoding controls

To assess if cell‐mediated PDGF‐BB expression impacted cellular behavior after prolonged pre‐incubation, cell proliferation was quantified via cell recovery from the modified collagen gels after 4 days of culture. Samples were analyzed at 4 days to exclude the effect of cellular confluency on cell behavior while determining the maximum period over which polyplex activity could be preserved in the presence of physiologically‐simulating conditions. As shown in Figure 3, the non‐preincubated unmodified polyplex samples and 20% GPP‐PEI/total PEI samples encoding PDGF‐BB exhibited elevated cell counts that were approximately 65% greater than the levels observed in the controls. After preincubation under physiologically‐simulating conditions for 5 days or longer, no significant difference in cell count was found in the unmodified polyplex samples, whereas cellular proliferation in the 20% GPP‐PEI/total PEI samples remained significantly elevated relative to the controls for up to a 7‐day preincubation. Proliferation in the 50% GPP‐PEI/total PEI samples remained elevated relative to controls for the longest period, with a significant increase of about 50% in cell counts even after a 10‐day preincubation. Additionally, the cell counts recorded in the 20% GPP‐PEI/total PEI were approximately 56% greater than those in the 50% GPP‐PEI/total PEI when the gels were not preincubated, but the count levels between the 20% and 50% samples were determined to be statically indistinguishable after either a 5‐day or a 7‐day preincubation. After a 10‐day preincubation, proliferation in the 50% GPP‐PEI/total PEI samples surpassed that recorded in the 20% GPP‐PEI/total PEI samples by approximately 23%. The cell counts recorded in the 50% GPP‐PEI/total PEI samples preincubated for 5, 7, or 10 days were statistically indistinguishable from one another. 14‐day preincubation studies were also conducted, however the results were not included because the samples exhibited no statistical difference from the controls and thus did not add any substantive information to the figure.

### 3.3 Quantification of PDGF‐BB‐mediated ECM remodeling

**Figure 4:**
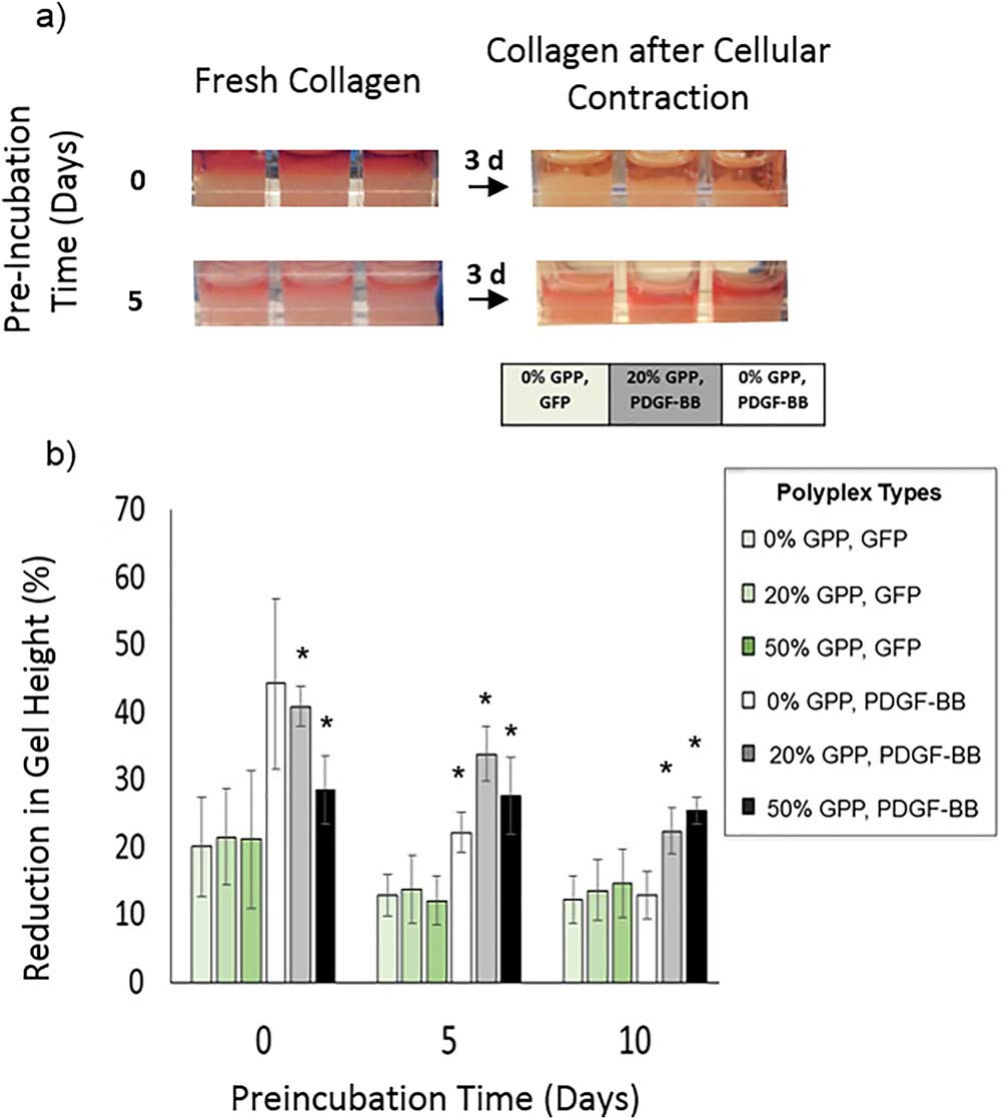
Collagen remodeling in the preincubated polyplex‐containing collagen gels in response to cell‐mediated expression of PDGF‐BB. Remodeling was monitored by measurement of the reduction in gel height (z), relative to the initial height in each gel, using ImageJ to assess gel images, 3 days after NIH/3T3 cells were seeded onto the gels. The data represent the mean ± SD of five separately prepared and analyzed samples. * denotes a statistically‐significant difference (*p* < .05) relative to the GFP‐encoding controls

A contraction assay was used to assess the level of ECM remodeling in response to cell‐mediated PDGF‐BB expression. As shown in Figure 4, gel heights shrank due to contraction at a significantly greater rate in all samples containing polyplex encoding for PDGF‐BB. Reflecting the PBGF‐BB quantification and PDGF‐BB‐mediated proliferation results, the non‐preincubated unmodified polyplex samples and 20% GPP‐PEI/total PEI samples exhibited the greatest reductions in gel height, deceasing approximately 20% more relative to the controls. After pre‐incubating the samples, no significant differences in gel height were observed in the controls versus the unmodified polyplex samples. However, the reduction in gel height remained significant in the 20% and 50% GPP‐PEI/total PEI samples for up to a 10‐day preincubation. Contraction was greatest in the 20% GPP‐PEI/total PEI sample when the sample was not preincubated, and contraction levels in this sample were indistinguishable after preincubation for 5 versus 10 days.

### 3.4 PDGF‐BB‐triggered migration in layered collagen gels

**Figure 5:**
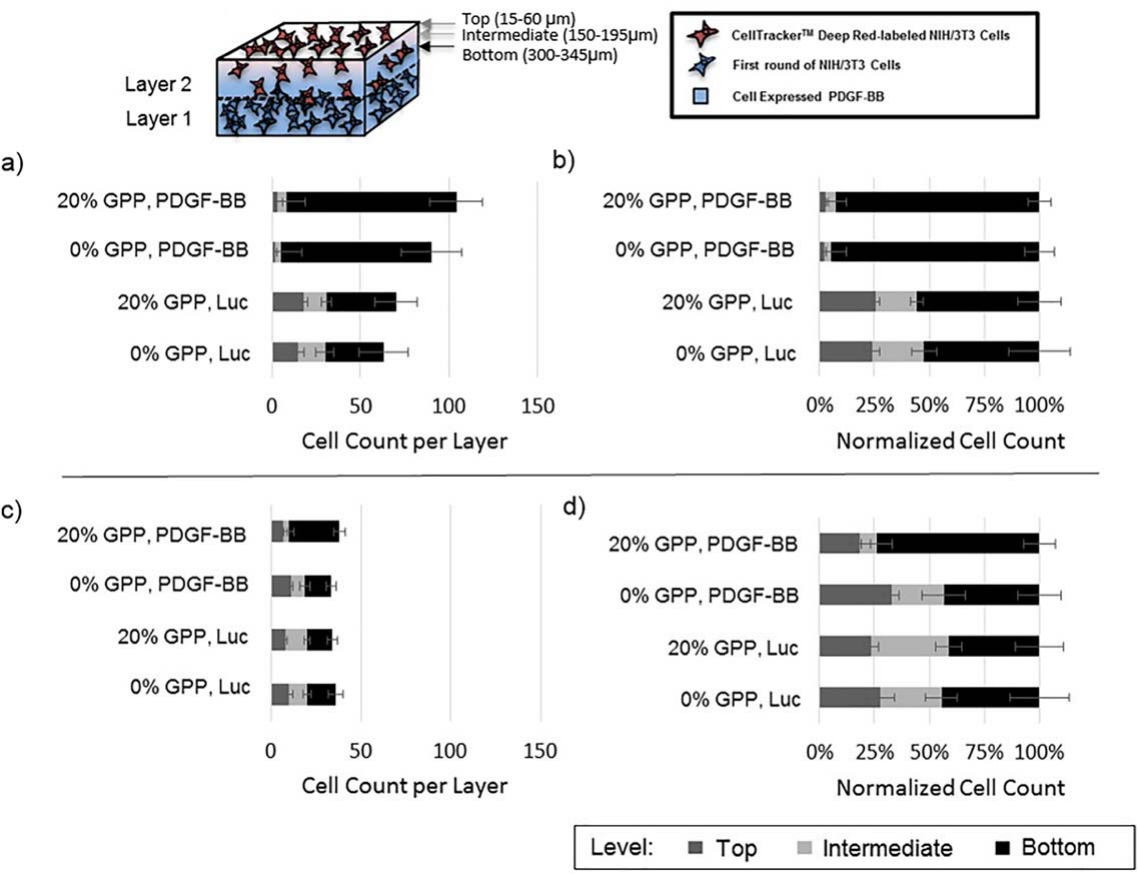
Cell migration in a collagen bi‐layer model in response to cell‐expressed PDGF‐BB was tracked using microscopy. The initial collagen layer (bottom layer; layer 1) was preincubated for 3 (a,b) or 10 (c,d) days before the addition of the next layer (layer 2). The data represent the mean ± SD in cell counts (left) and cell counts normalized by the total number of analyzed cells per gel (right) of four separately prepared and analyzed samples. Cells were allowed to migrate for 48 hours prior to analysis

To determine if cell‐mediated PDGF‐BB expression triggered chemotaxis, layered collagen gels were constructed. The first layer (layer 1) contained polyplex encoding for PDGF‐BB (or luciferase, as a control), and cellular ingrowth and gene transfection were allowed to occur within this layer for a period of 2 days. Subsequently, a second collagen layer (layer 2) was added, and additional cells containing a tracking dye were plated atop this fresh collagen (Figure 5a). The labeled cells in the second layer were allowed to migrate in response to PDGF‐BB expressed by cells in the first layer, and the extent of cell migration toward the bottom layer was quantified via fluorescence microscopy. As shown in Figure 5, the length of time the first layer was preincubated directly impacted cellular migration. Specifically, in samples in which the first layer was preincubated for 3 days, all samples containing the PDGF‐BB‐encoding polyplex exhibited accelerated migration as compared to controls in which luciferase‐encoding polyplexes were used. In these samples, about 90% of the analyzed cells migrated 300 µm toward the bottom layer when the first layer contained PDGF‐BB‐encoding polyplexes, whereas only ∼50% of the cells migrated as far with the luciferase‐encoding polyplexes. When the first layer gel was preincubated for a 10‐day period, approximately 44% of cells migrated to the bottom level in both the luciferase‐encoding polyplex samples and the unmodified polyplex samples, whereas almost 75% of the analyzed cells had migrated to the bottom layer in the 20% GPP‐PEI/total PEI PDGF‐BB‐encoding samples. A significantly greater amount of cells were also observed in the intermediate level of the gel in the unmodified polyplex samples relative to the GPP‐modified samples.

### 3.5 In vitro wound model

**Figure 6:**
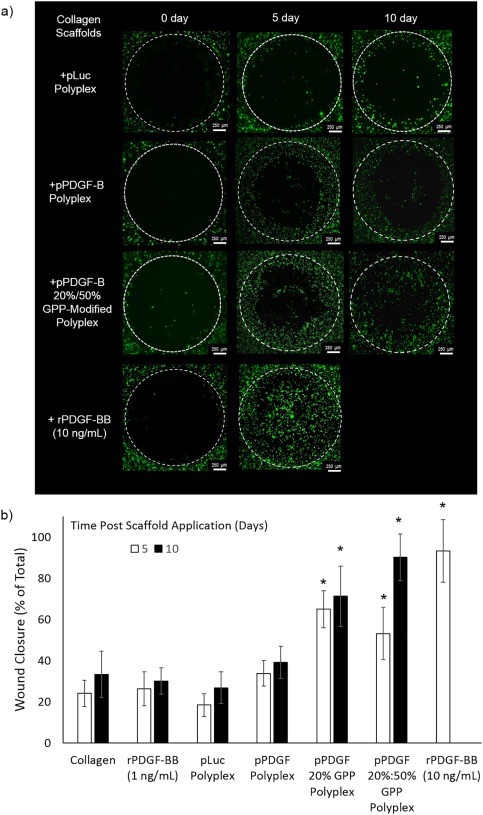
In vitro wound model. Defects in cell‐seeded collagen gels were filled with collagen scaffolds modified with rPDGF‐BB, polyplex encoding for luciferase, or polyplex encoding for PDGF‐BB, and subsequent defect invasion or “wound closure” was monitored via microcopy. Representative images (a) were analyzed via ImageJ to quantify wound closure (b). The data represent the mean +/‐SD of three separately prepared and analyzed samples. * denotes a statistically‐significant difference (*p* < .05) relative to the luciferase‐encoding controls

To culminate the studies, the ability to achieve enhanced wound closure via cellular responses triggered by cell‐expressed PDGF‐BB was examined in an *in vitro* wound model (Figure 6). Cellular invasion of uniform wounds created in collagen after application of DNA‐modified collagen scaffolds or collagen embedded with rPDGF‐BB was monitored via calcein AM staining. In the samples in which a scaffold containing polyplex encoding PDGF‐BB was administered, accelerated cellular invasion was noted, particularly in the GPP‐modified samples. Specifically, the wound closure in the unmodified sample encoding for PDGF‐BB was only statistically different from closure in the luciferase‐encoding polyplex control, whereas it was not statistically distinguishable from pure collagen or low rPDGF‐BB‐containing controls (1 ng/mL PDGF‐BB). After 5 days, the 20% and 20%/50% GPP‐modified samples achieved similar levels of wound closure (approximately 65% and 53%, respectively), but these samples did not achieve the same levels of closure as the high rPDGF‐BB dosage control (10 ng/ml PDGF‐BB). After an additional 5 days (10 days post‐defect), a significant increase in percentage of wound closure was only recorded in the GPP‐modified samples, with the 20%/50% GPP‐modified sample achieving the same level of wound closure as the high dosage rPDGF‐BB control. Studies with 50% GPP‐modified samples were also initiated, but these samples were terminated early due to the low levels of observed wound closure within the monitoring period (Supporting Information Figures S1 and S2). Additionally, a similar in vitro wound defect model employing prelabeled cells was used to assess wound closure at early time points, and these experimental demonstrated that wound closure did not occur in any samples during the initial 3 days (Supporting Information Figure S1).

## 4 Discussion

The inherent complexity and hostility of the chronic wound environment has greatly complicated treatment, causing the failure of most monotherapies containing just GFs or biomaterial‐based scaffolds.^3^, ^37^, ^59^ Chronic wounds exhibit a self‐sustaining, out‐of‐control inflammatory response in which excessive protease activation impairs cellular infiltration and causes the rapid degradation of GFs and other molecules essential for the reparative process.^3^, ^5^ The addition of GF through topical or sustained applications has been shown to enhance repair in several human studies, demonstrating its potential for modifying the wound environment; however, the modest degree of clinical success with these approaches underscores significant deficiencies in the way GFs are presented. GF therapies are reliant on repetitive, extraphysiological doses, increasing the likelihood of off‐target effects and oncogenic responses. GF gene therapies, particularly when delivered via a biomaterial, exhibit benefits in protein stability, protein bioactivity, sustained release, and cost. The potential to deliver multiple genes with relative ease and the spatiotemporal control achievable though promoter choice/vector design are also extremely advantageous. Moreover, gene delivery better mimics endogenous repair, allowing host cells to orchestrate GF production, microlocalization, and activity, which are particularly crucial in mitigating the pathophysiology of a chronic wound.

In our work, we have shown that CMP‐mediated gene delivery has compelling advantages for overcoming the obstacles that have prevented the translation of genetic therapeutics in wound repair. CMP‐based gene transfer reduces the dangers of therapeutic escape and abrogates concerns of low activity in protein/serum‐rich environments. In particular, we demonstrated previously that GPP‐modification of PEI polyplexes greatly enhanced control over both the extent and duration of transgene expression through utilization of the reversible, serum‐stable affinity between CMPs and natural collagen. Enhanced control and activity were demonstrated both in vitro^34^ and in vivo, using a murine subdermal repair model.^56^ Additional studies concluded that these improvements were the result of superior serum‐stability and endocytic trafficking driven by increases in polyplex affinity for collagen and the natural process of collagen remodeling. While other reports have utilized collagen remodeling/proteolytic sensitivity to achieve cell‐triggered release, CMP‐modification provided a tool for direct integration of both release and endocytic uptake with collagen remodeling, while providing enhanced serum‐stability.

Having demonstrated the ability to integrate gene delivery with collagen remodeling, our objective in this work was to incorporate our DNA/collagen system into a wound environment and demonstrate its benefits for promoting key wound repair activities. Cell invasion, proliferation, and ECM remodeling are prerequisites for both wound healing and the initiation of gene transfer activity from the GAMs, and therefore PDGF‐BB, which is capable of stimulating these cellular responses, was a natural target. Through modification with a CMP containing an established, GPP‐rich sequence, we were able to identify significant improvements in both PDGF‐BB expression and activity. GPP modification substantially expanded the length of time the DNA/collagen gels could be exposed to physiological‐simulating conditions (37°C in serum‐supplemented media), from 0 days to 10 days, and still observe enhanced PDGF‐BB expression. While MMP concentrations are elevated in the wound environment, no MMPs or wound‐relevant proteases were added during the pretreatment step, based on studies showing localization of MMPs in the cellular microenvironment at the wound edge or immediate tissues.^64^ Utilizing preincubation to simulate the environment of an applied collagen‐based dressing before cell invasion, this study suggests that GPP modification can be used to prolong the activity of DNA polyplexes during the process of cellular invasion, which often occurs over a period of weeks to months in chronic wounds. Varying the display of GPP on the polyplex from 20% GPP‐PEI/total PEI to 50% GPP‐PEI/total PEI expanded the period the gels could be exposed to physiological‐simulating conditions from 5 days to 10 days, suggesting that GPP display could also be used to tailor the extent and period of PDGF‐BB expression as required. Additional studies have also shown varying CMP sequence allows for further tailoring of retention/release, suggesting additional tuning could be achieved with relative ease. For example, it was demonstrated that lengthening the CMP sequence (GPO_7_ vs. GPO_10_) could be used to decrease the CMP‐collagen initial dissociation index (IDI) by 1.7‐fold and expand retention/release of a GPP‐modified polymer from approximately 7 days to over 12 days.^67^

Our studies also highlighted the benefits of GF gene versus GF protein delivery, and demonstrated in particular the mechanisms by which GPP‐modification of gene vectors amplifies these benefits. For instance, we demonstrated the ability to trigger cellular responses similar to those observed in rPDGF‐BB/collagen systems, yet with three orders‐of‐magnitude lower levels of local PDGF‐BB expression than the levels of rPDGF‐BB delivered in GF‐collagen therapies.^6^, ^8^, ^37^, ^43^, ^58^ DNA/collagen gels containing PDGF‐BB‐encoding polyplex were able to trigger cellular behaviors not observed in controls with luciferase encoding polyplex. Cell proliferation was increased by up to 65%, gel contraction was increased by as much as twofold, and cell migration was triggered via cell‐produced PDGF‐BB gradients. When PDGF‐BB‐encoding polyplexes were used in collagen bi‐layer migration studies, as many as 90% of cells had migrated 300 µm into the gel within 48 hr post cell seeding. Furthermore, cells were visible at this level (300‐345 µm) within 24 hr post cell seeding, and given that the typical speed of NIH/3T3 cell migration in collagen is less than one cell diameter per hour, the determined speed of these cells (12.5 µm/hr) indicates both the directionality and consistency of their migration.^31^ Our findings accentuate the importance of cell‐mediated expression and micro‐localization encouraged by efficient gene delivery, and show how gene delivery can be used to overcome the high costs and safety concerns associated with high doses of GFs. Consistent with the enhanced levels of PDGF‐BB detected in samples containing GPP‐modified, PDGF‐expressing polyplex, GPP‐modification was found to stabilize the systems even after prolonged periods of preincubation. For example, increases in proliferation were observed after a 10‐day preincubation, whereas no increases were observed in any of the unmodified samples preincubated for this length of time. Collagen remodeling and chemotaxis were also enhanced for up to a 10‐day preincubation in GPP‐modified samples, versus only a 5‐day preincubation in unmodified samples. The extent of GPP‐modification was also found to expand other observable cellular responses, underscoring the flexibility CMP‐modification provides. For example, cell counts in 20% GPP‐PEI/total PEI samples were increased by up to 65% relative to luciferase controls, and these levels remained statistically greater than the controls for up to a 7‐day preincubation. Alternatively, cell counts in the 50% GPP‐PEI/total PEI samples preincubated for 5 or 7 days were consistently about 50% greater than those in the luciferase controls, and these counts were statistically indistinguishable from those recorded in the 20% GPP‐PEI/total PEI samples. Cell counts remained significantly enhanced for up to a 10‐day preincubation in the 50% GPP‐PEI/total PEI samples. The fact that increased proliferation was not observed for the same times periods over which enhanced chemotaxis and remodeling were observed is consistent with the finding that higher concentrations (>5 ng/ml) are required to promoted mitosis, whereas lower concentrations (<1 ng/ml) are sufficient for migration.^2^

The benefits of GPP‐mediated *PDGF‐B* gene delivery also translated in a 3‐D wound model where wound closure was defined as the point when the average cell density in the defect matched the average cell density in the area surrounding the defect. Collagen‐based scaffolds containing PDGF‐BB‐encoding polyplex promoted increased invasion and elevated cell densities within the defects within 3 days (unpublished data). Mirroring the results observed in the preincubation studies, GPP‐modification appeared to preserve the activity of the scaffolds in environments containing nucleases and serum proteins at physiological temperatures. Scaffolds with the 20% GPP‐modified polyplex had wound closure rates 2.3‐fold and 1.6‐fold faster than those observed in the empty collagen scaffolds and in the scaffolds containing unmodified PDGF‐BB‐encoding polyplex, respectively, over a 5‐day closure period. By day 10, wounds containing the 20% GPP‐modified polyplex scaffolds had induced nearly a 2.1‐fold and 2‐fold increase in wound closure compared to wounds in which empty collagen and unmodified polyplex scaffolds were applied. The relative rate of closure was comparable to that achieved using in other in vitro wound models^37^ as well as in vivo models^8^, ^28^ of soft tissue wounds. For example, the application of PDGF‐BB encoded by plasmid or adenovirus DNA vector embedded in collagen matrix induced a 2.5‐fold increase in wound closure in 6‐mm or 8‐mm ischemic skin wounds in rabbits, relative to a negative control.^8^, ^28^ To achieve the same effect in our wound model with PDGF‐BB, an order of magnitude increase in rPDGF‐BB exposure was required. The greater dosage not only necessitates greater treatment costs, but also increases the danger of off‐target responses, as PDGF‐BB leakage from collagen‐gels and sponges is a major problem in protein‐based delivery.^68^ Moreover, when rPDGF‐BB was applied in the wound model at the same concentration as that expressed in the GPP‐modified collagens (1 ng/ml), wound closure was indistinguishable from the empty collagen control, further supporting the fact that efficient gene delivery allowed dramatically reduced dosages. The identical levels of overall wound closure via gene delivery were achieved in this model through application of an 8:2 (m/m of DNA in the 20% vs. 50% GPP‐modified polyplex) mixture of 20%/50% GPP‐PEI/total PEI polyplex. Five days after application, the percent of wound closure was identical to that in the 20% GPP‐PEI/total PEI sample, but while the 20% sample plateaued at approximately 75% wound closure, the combined polyplex sample achieved over 90% wound closure, the same as the high dosage rPDGF‐BB control. These results highlight the versatility of CMP‐modification, where both CMP sequence and display can be used to tailor not only release, but also the extent and duration of GF expression. While these outcomes have been demonstrated here in a simplified wound model, the results support the potential utility of localized gene delivery over protein delivery in the more general context.

## 5 Conclusion

GF gene therapies may have the potential to overcome the innate incompatibility many current GF therapies have with the wound bed, through promoting on‐demand GF production that better mimics endogenous repair responses by allowing host cells to orchestrate sustained GF expression, microlocalization, and activity. CMP‐modification of DNA carriers, particularly PEI polyplexes, has been demonstrated by our labs and shown to enable the hijacking of not only the cellular machinery needed to express GFs (or any gene of interest), but also the cellular mechanisms inherent in the natural process of collagen remodeling to achieve cell‐triggered delivery and enhanced endocytic uptake. In this work, GPP‐modification was shown to enhance expression of functional GF (PDGF‐BB) and trigger essential cell behaviors associated with wound repair. Furthermore, CMP‐mediated gene delivery achieved similar levels of wound closure as the levels reached when an order of magnitude more rPDGF‐BB was applied. The fact that a combination of 20% and 50% GPP‐PEI/total PEI polyplexes could be used to tailor this response highlights the versatility of this approach, and identifies additional tuning factors which have yet to be fully explored. Both the tunablity of this method and its ability to hijack collagen remodeling, a process that already occurs in excess in wounds, make this an ideal method for achieving efficient GF delivery in the wound bed.

## Conflict of interest

The authors have declared no conflicts of interest exist.

## Supporting information

Supporting Information 1
