# Supplementary material for: Integration of growth factor gene delivery with collagen‐triggered wound repair cascades using collagen‐mimetic peptides
Source: Bioeng Transl Med. 2016 Oct 19;1(2):207–19. doi: 10.1002/btm2.10037 (PMC5125401; doi:10.1002/btm2.10037)
Supplement: Supplementary file 1 — Supporting Information 1 [file BTM2-1-207-s001.pdf]

SUPPLEMENTAL INFORMATION

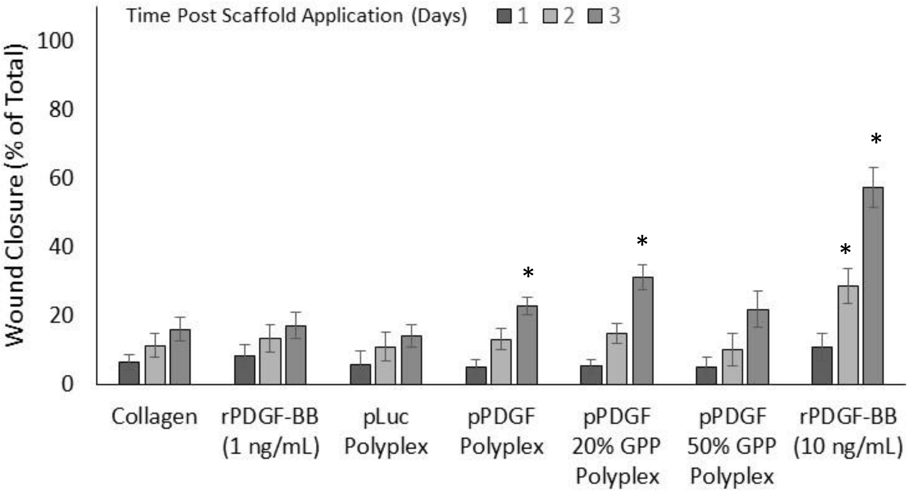

Figure S1. *In vitro* wound model wound closure at early time points. To evaluate initial closure, the same defect model was implemented with NIH/3T3 cells pre-labeled with CellTracker^{TM} Deep Red. As previously described, defects in cell-seeded collagen gels were filled with collagen scaffolds modified with rPDGF-BB, polyplex encoding for luciferase, or polyplex encoding for *PDGF-B*, and initial defect invasion was monitored via microcopy. The data represent the mean +/-standard deviation of 3 separately prepared and analyzed samples. * denotes a statistically-significant difference ($p<0.05$) relative to the luciferase-encoding controls.

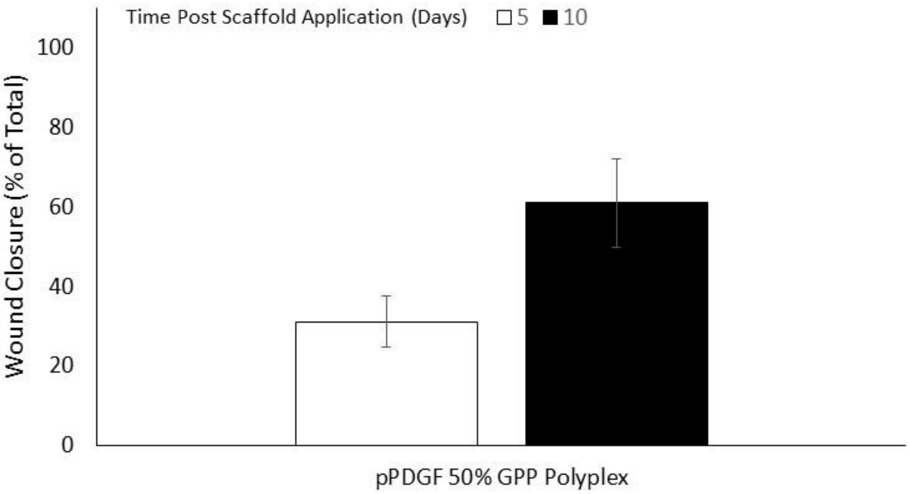

Figure S2. *In vitro* wound model. As previously described, defects in cell-seeded collagen gels were filled with collagen scaffolds modified with polyplex encoding for *PDGF-B*. Defect invasion was monitored via microcopy and the effect of GPP-modification was studied. The data represent the mean +/-standard deviation of 2 analyzed samples.
